# Six-Week Protocol for Two-Stage Exchange Arthroplasty in a High-Risk Population

**DOI:** 10.1016/j.artd.2026.101987

**Published:** 2026-03-06

**Authors:** Alexander Mass, Myrla Sajo, Eric Silverstein

**Affiliations:** a Frank H. Netter MD School of Medicine, Quinnipiac University, North Haven, CT, USA; b Trinity Health of New England at Saint Francis Hospital, Hartford, CT, USA; c Connecticut Joint Replacement Institute at Saint Francis Hospital, Hartford, CT, USA

**Keywords:** Infection, Two-stage exchange, Revision, Mega-prostheses

## Abstract

**Background:**

Periprosthetic joint infection (PJI) remains a devastating and serious complication following total joint arthroplasty. The gold standard for infection eradication is a 2-stage exchange, yet there remains no consensus on optimal timing, antibiotics regimen, holidays, or aspirations. This study aims to evaluate a 6-week 2-stage exchange protocol in treating PJI for hip and knee arthroplasties in a high-risk population.

**Methods:**

A retrospective review was conducted of patients who underwent 2-stage revision for PJI between January 1, 2018, and December 31, 2022. Patients met Musculoskeletal Infection Society (2018) criteria for PJI and were treated with a standardized 6-week 2-stage exchange protocol. The primary outcome was infection eradication, defined as the absence of reoperation. Secondary outcomes included infecting organisms, culture positivity rates, and outcomes in patients with mega-prostheses.

**Results:**

Seventy-five patients (77 joints), of which 42 joints (55%) were mega-prosthetics met criteria, 42% demonstrated pathology-proven osteomyelitis. Infection eradication was achieved in 88% of cases at a minimum of 1 year. Nine patients (8/9 were mega-prosthetics) returned for reoperation. The culture positivity rate was 69%, with *Staphylococcus aureus* and coagulase-negative *Staphylococcus* being the most common pathogens. Thirty-one patients (40%) remained on suppressive antibiotic therapy for 1 year (27/31 were mega-prosthetics).

**Conclusions:**

A shortened multidisciplinary 6-week protocol for 2-stage exchange revisions is a viable, reliable, and effective strategy for managing PJI. Despite a high-risk population with osteomyelitis and mega-prostheses, outcomes were similar or better than traditional approaches. This shortened protocol demonstrated high eradication rates while potentially reducing resource utilization, costs, and patient’s burden.

## Introduction

Two of the most common surgical procedures are total knee arthroplasty (TKA) and total hip arthroplasty (THA), both of which are expected to substantially increase by 2030 [1]. Projections estimate the number of revision total knee arthroplasty (rTKA) are to increase between 43% and 70% and the number of revision total hip replacements are to increase between 78% and 182% by 2030 from 2014 levels [2].

One common complication of joint arthroplasty is periprosthetic joint infection (PJI), with an incidence rate of 1%-2% [3]. The approximate number of infected THAs and TKAs in 2017 was 11,985 and 18,400, respectively. This is expected to increase as the number of arthroplasties increase, and by 2030 the projected number are 25,928 and 40,096 respectively which is estimated to cost $1.85 billion [3].

The gold standard treatment for revision arthroplasty in the setting of chronic periprosthetic infection remains 2-stage revision [[4], [5], [6], [7]]. Two-stage revision is the primary method for treating chronic PJI in North America, including the United States [8]. Current literature suggests the effectiveness of 2-stage arthroplasty for infection ranges from about 72% to 95% with an average around 85% [4,9]. However, the timing and components used in treating chronic periprosthetic infections are often highly variable and there is no consensus on an ideal protocol with antibiotic treatment, timing, holidays, or aspirations [10]. At the 2018 Proceedings of International Consensus on Orthopaedic Infections, no consensus was reached regarding the optimal timing for reimplantation due to a lack of conclusive evidence [11]. This leaves the decision in timing for reimplantation at the discretion of the healthcare team. Thus, there remains controversy in the protocol for 2-stage revision of TKA and THA.

Based on Infectious Disease Society of America guidelines, patients with chronic prosthetic joint infection who undergo 2-stage exchange arthroplasty should receive 4 to 6 weeks of pathogen-specific intravenous (IV) or highly bioavailable oral antimicrobial therapy. [12]. There is also an antibiotic-free period of 2 to 8 weeks prior to delayed reimplantation. This strategy is used frequently in the United States.

In Europe, there is a favorable outcome with reimplantation within 2 to 6 weeks while systemic antimicrobials are still being administered if the infection is not due to methicillin-resistant *Staphylococcus aureus* (MRSA), enterococci, multidrug-resistant gram-negative organisms [13]. In a study by Winkler et al., 2-stage exchange with a short interval (<4 weeks) has a similar outcome than with a long interval (>4 weeks), when highly active antibiotic therapy is used [14]. Antimicrobial therapy was not stopped before prosthesis reimplantation.

The goal of having a shorter interval between the first stage and second stage is to reduce pain and psychological impact, return the patient as quickly as possible to work, provide a feeling of “normality” in addition to reducing cost and burden to the healthcare system. Precisely the reason why one-stage arthroplasties have been gaining traction [[15], [16], [17], [18], [19], [20]].

By reducing the amount of time in between the interval, patients have less time with limited mobility of the joint thus decreasing patient impact. The primary purpose of the study is to evaluate the outcome of a 6-week protocol for 2-stage arthroplasty in a high-risk population. A failure is defined as a return to the operating room for signs or symptoms consistent with infection within 1 year following the 2-stage procedure. Our population is defined as high risk given their medical comorbidities, prior surgical history, and elevated risk for chronic osteomyelitis.

## Methods

Institutional review board approval was obtained to perform a retrospective review of our 6-week protocol for treating chronic periprosthetic hip and knee infections. The surgical timeline was conducted between January 1, 2018, and December 31, 2022. The patients were either male or female above the age of 18. There was no exclusion based on gender, and anyone under the age of 18 was excluded. The patients were all collected from a single institution with our own database, which is certified by the American Academy of Orthopaedic Surgeons. The data were gathered by utilizing a retrospective cohort. The inclusion criteria included revisions being performed by 3 surgeons following the 6-week protocol. These patients were chosen after satisfying the requirements for chronic periprosthetic infection per the Musculoskeletal Infection Society (MSIS) (2018) criteria [21]. Nearly all the patients had a preoperative erythrocyte sedimentation rate (ESR), C-reactive protein (CRP), and white blood cell count. In addition, nearly all had a preoperative aspiration evaluating for cell count, absolute number of nucleated cells (including percentage of cell type, eg, polymorphonuclear neutrophils), culture (monitored 14 days), and crystals. All patients met the MSIS (2018) criteria for PJI. Patients who did not satisfy the preoperative criteria for PJI because of low synovial fluid count or a dry tap had intraoperative confirmation such as the presence of a sinus tract, isolation of the same organism at least twice in tissue samples and histopathologic findings. After determining that the patient was infected, we followed a strict protocol by performing a 2-stage revision.

For knees, the patient had a first-stage explantation, thorough debridement, incision and drainage, and placement of a static antibiotic-loaded spacer with resorbable antibiotic beads. In most cases, the spacer consisted of Ender’s rods placed intramedullary and held together in the joint with additional cement. The antibiotic rods (total of 1 bag of cement) were formed by placing the Ender’s rods into silicon tubing that created a 13 mm diameter. A total of 5 bags of Simplex cement (Stryker, Mahwah, NJ, USA) was used with 2 g of vancomycin and 1.2 g of tobramycin per bag for the entire construct. The beads were placed both inside the bone prior to placement of the rods and within the joint. We utilized Stimulan (Biocomposites, Keele, United Kingdom) (10cc which creates 25cc of beads) premixed with 1 g of vancomycin and 1.2 g of tobramycin. A minimum of 5 cultures was taken intraoperatively. These always included the following:

1. Synovial fluid swab
2. Anterior synovium (tissue)
3. Fat pad (tissue)
4. Distal femur (tissue)
5. Proximal tibia (tissue)

Any additional suspicious areas were cultured if deemed necessary.

For treatment of hips, the first-stage explantation was followed by a thorough debridement, incision and drainage, and placement of a mobile spacer with resorbable beads. The spacer consisted of the Zimmer Echo Bi-metric cemented collared stem (Zimmer Biomet Holdings, Inc., Warsaw, IN, USA) wrapped with antibiotic-loaded cement around the proximal metaphyseal flare. A long-stem variant (Biomet Bi-metric Meta-400) (Zimmer Biomet Holdings, Inc., Warsaw, IN, USA) was used if a proximal femoral resection was necessary. Resorbable antibiotic beads were loaded within the femoral canal prior to the stem placement (Stimulan as noted above). For the acetabulum, a cemented all-polyethylene Biomet Freedom cup or standard Biomet Freedom polyethylene (Zimmer Biomet Holdings, Inc., Warsaw, IN, USA) which was etched on the backside with a Midas burr was used. These were cemented in place with antibiotic cement. In most cases, a total of 3 bags of Simplex cement was used, premixed with 2 g of vancomycin and 1.2 g of tobramycin per bag. A minimum of 5 cultures were taken intraoperatively. These always included the following:

1. Synovial fluid swab
2. Hip capsule and synovium (tissue)
3. Proximal femur (tissue)
4. Behind polyethylene (swab)
5. Acetabulum (tissue)

Any additional suspicious areas were cultured if deemed necessary.

**Figure 1:**
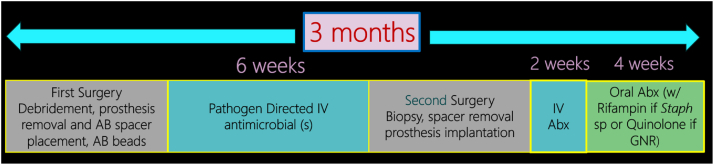
Treatment protocol for the 2-stage exchange arthroplasty. AB, antibiotic; Abx, antibiotics; GNR, gram-negative rods.

After completing either the first-stage explantation of the hip or knee, the cultures were followed by a dedicated musculoskeletal infectious disease physician. These cultures were monitored for a total of 2 weeks. All patients received broad-spectrum antibiotics only after intraoperative tissue sampling. The choice of empiric IV antibiotics was based on the previous preoperative culture result and patient’s allergy profile. We would eventually change to a targeted antibiotic therapy as soon as cultures were finalized. The patients were then maintained on IV antibiotics for 6 weeks. Rifampin was never prescribed in the implant-free interval. We did not withhold antibiotics on any patient and did not reaspirate. The patient was then brought back to surgery after the 6 weeks for a second-stage revision operation. During the second operation, a minimum of 5 cultures were done. Preoperative antibiotic prophylaxis was not withheld prior to intraoperative tissue sampling during the second stage. The same sites were used or modified as necessary. If all the gram stains were negative, we proceeded with the reconstruction. We did not utilize frozen sections from pathology. This was based on prior experience and poor reliability. Following the second-stage reconstructions, the patients were placed back on IV antibiotics for a minimum of 2 weeks until the cultures were finalized. If the cultures were negative, 4 additional weeks of oral antibiotics were given for a total of 6 weeks postoperative antibiotics. However, if at least 2 of the 5 cultures were positive with the same bacteria, then the patient was treated for a minimum of 3 months of antibiotics following the surgery. This consisted of 2 weeks IV and the remainder was oral. If a patient had a mega-prosthesis with at least 2 positive second-stage cultures, lifelong suppressive antibiotics was utilized. After the second-stage surgery, we employed oral antibiotics with antibiofilm activity such as rifampin combined with another companion drug against Staphylococcus infection and a fluoroquinolone against Gram-negative infection if susceptible. The treatment protocol is summarized in Figure 1.

We evaluated the patients by determining the pathogenic organism responsible for the infection, antibiotic sensitivities, clinical progress, blood work, and whether they displayed any signs or symptoms compatible with infection. These patients were monitored for a minimum of 1 year from the time of the second-stage revision surgery. Return to the operating room for any reason or readmission was recorded. We considered a success any patient that remained disease free both in the joint and systemically. In addition, they demonstrated improvement in their joint replacement compared to the preoperative status.

## Results

Our database captured a total of 75 patients with 77 chronically infected total joint arthroplasties based on the MSIS (2018) definition of prosthetic joint infection. Two patients were treated for bilateral infections. Fifty-two were knees (67.5%) and 25 were hips (32.5%). The patient population was diverse. The mean age of the patients was 66.5 years. The mean American Society of Anesthesiologists Physical Status score was 2.8 and the mean body mass index was 31.8 kg/m^2^. The most common infecting organism was *Staphylococcus aureus* (23%), of which one-third were MRSA. The second most common infecting organism was coagulase-negative staphylococcus (18%) (Table 1). The cultures from the first-stage resection had a positivity rate of 69% (Table 2). This required a minimum of 2 positive cultures with the same organism in concordance with MSIS (2018) criteria.

**Table 1:** Infecting organisms.

Organism	Number (n)	Percentage (%)
*Staphylococcus aureus*	18	23
Methicillin-resistant *Staphylococcus aureus* (MRSA)	6	8
Methicillin-susceptible *Staphylococcus aureus* (MSSA)	12	15
Coagulase negative *Staphylococcus*	14	18
Methicillin-resistant	5	6
Methicillin-susceptible	9	12
*Enterococcus* sp.	1	1
*Streptococcus* sp.	9	12
Gram-negative bacteria	7	9
*Cutibacterium acnes*	4	5
*Candida* sp.	1	1
Polymicrobial	4	5

**Table 2:** Culture positivity.

Culture positivity	Percentage (%)
Positive	69
Negative	31

Of the 77 surgeries performed, 68% (n = 52) were for the knee and 32% (n = 25) for the hip. The most common second-stage knee reconstructions included a revision TKA (n = 22) and a distal femoral replacement (n = 27). The second-stage hip reconstructions included revision THA in 11 patients and a proximal femoral replacement in 14 patients (Table 3).

**Table 3:** Reconstruction type.

Type of reconstruction	Number (n)	Percentage (%)
Knee	52	68
TKA	22	29
Distal femoral replacement (DFR)	27	35
Fusion	2	3
Total femoral arthroplasty	1	1
Hip	25	32
THA	11	14
Proximal femoral replacement (PFR)	14	18

**Figure 2:**
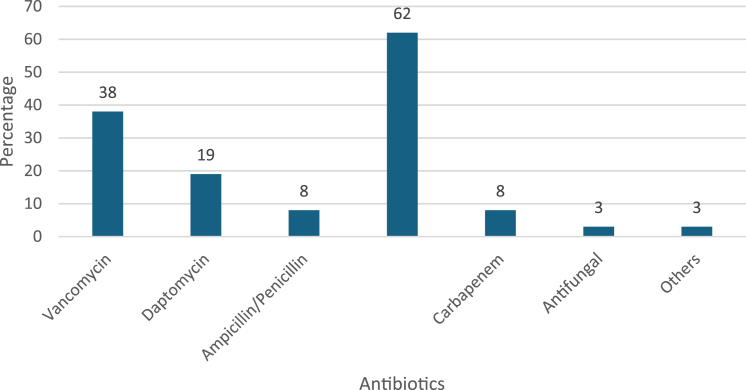
Antimicrobials used after the first-stage surgery.

With regard to antimicrobial utilization, the majority of the patients received cephalosporin, namely cefazolin, ceftriaxone, and cefepime for which ceftriaxone comprised 65% (Fig. 2). Vancomycin was only used among 38% of patients. The proportion of patients who had combination of antimicrobials between the 2 stages is 39%.

We had 4 *Cutibacterium acnes* prosthetic joint infections, and they received either ceftriaxone, penicillin, or vancomycin depending on their allergy profiles. The antibiotics incorporated in the cement are vancomycin and tobramycin except in the presence of vancomycin allergy for which daptomycin was used. One of our patients had a preoperative diagnosis of Candida prosthetic joint infection so vancomycin, tobramycin, and amphotericin B were mixed with the cement. They received micafungin and did not require any surgery within 1 year of implantation of a distal femoral replacement and was placed on suppressive antibiotic therapy.

Following the second-stage reconstructions of all patients, 9% (7 of 77 joints) had positive cultures (Table 4). One of the cases had concordant cultures and remained on suppressive antibiotic treatment (SAT), whereas the other 6 were discordant. Among those who had positive cultures on reimplantation, only 1 patient was not placed on SAT. Overall, 40% (n = 31) of the patients were placed on SAT for 1 year (Table 5). Only 4 of those patients had standard revision implants as opposed to a mega-prosthesis.

**Table 4:** Positive cultures on second-stage reimplantation.

Culture status at reimplantation	Number (n = 77)	Percentage (%)
Total positive cultures	7	9%
On SAT (suppressive antibiotics)	6	-
Not on SAT	1	-
Concordant cultures	1	-
Discordant cultures	6	-

**Table 5:** Proportion of patients on SAT (suppressive antibiotic treatment) for 1 year.

SAT for 1 year	Number (n = 77)	Percentage (%)
Yes	31^a^	40
No	46	60

a Only 5 out of 33 patients with THA and TKA [3] are on SAT – 15%.

Of the 77 surgeries performed, 54% of the patients (n = 42) had a mega-prosthetic reconstruction. By evaluating those patients only, 57% (24/42) were placed on suppressive antibiotics (Table 6). We reviewed our return to the operating room for any surgery within 1 year from the second-stage revision and found a 12% rate of return (9 patients). Of the 9 patients who returned for additional surgery, 8 had a mega-prosthesis and 1 had a revision TKA. Eighty-eight percent of patients did not require surgery after the revision for a minimum of 1 year. Five out of the 9 patients who returned for surgery were on suppressive antibiotics and had mega-prosthesis (Table 7). Among those with positive culture on reimplantation, only 1 patient with a total femoral arthroplasty underwent surgery within a year. We also evaluated osteomyelitis diagnosed from histopathology during the first- or second-stage surgery in most but not all patients. We demonstrated that at a minimum 42% of patients (32/77) had pathology-proven osteomyelitis. We did not have data on 23% (18/77). Fifty-two percent (22/42) who were reconstructed with a mega-prosthesis had pathology-proven osteomyelitis (Table 8). There are 4 patients who had pathology-proven osteomyelitis who had surgery within 1 year. All of them had mega-prosthesis. Approximately 55% (42/77) of all patients had at least 1 surgery prior to our surgical intervention and 29% (22/77) had 2 or more surgeries (Table 9).

**Table 6:** Proportion of patients with mega-prosthesis who are on SAT (suppressive antibiotics).

Mega-prosthesis status	Number (n = 77)	Percentage (%)
Total mega-prosthetics	44	55
On SAT	26	31
Not on SAT	18	23

**Table 7:** Return to the operating room for any surgery within 1 year of second-stage revision.

Return to operating room within 1 year	Number (n = 77)	Percentage (%)
Any surgery within 1 year	9^a^	12
Mega-prosthesis	8	
TKA	1	
No surgery within 1 year	68	88

a 5 of 9 patients who had surgery within 1 year were on suppressive antibiotics.

**Table 8:** Proportion of patients with pathology-proven osteomyelitis.

Osteomyelitis status	Number (n = 77)	Percentage (%)
Osteomyelitis	32^a^^,^^b^^,^^c^	42
No osteomyelitis	27	35
No data	18	23

a 22 patients with a mega-prosthesis had pathology-proven osteomyelitis.

b 15 patients with a mega-prosthesis and osteomyelitis are on SAT.

c 4 patients with a mega-prosthesis and osteomyelitis had surgery within 1 year.

**Table 9:** Number of surgeries prior to consultation.

Number of surgeries before consult	Number (n = 77)	Percentage (%)
0	35	45
1	20	26
≥2	22	29

## Discussion

Chronic PJIs continue to be difficult to treat and there is no clear consensus in the literature regarding the most effective procedure to eradicate them. In some countries, a single-stage revision arthroplasty is common practice, whereas in the United States, the gold standard remains a 2-stage arthroplasty. It has yet to be determined what is the most effective treatment interval between the first- and second-stage operation [22]. In addition, there has been no standardization regarding antibiotic coverage, route, and duration. For this reason, we began standardizing our approach by investigating a shorter time interval between stage 1 and stage 2. We utilized a multidisciplinary model and regimented surgery to improve outcomes.

The results suggest that our approach is as effective as the more commonly used practice of a longer time interval between stages. On average, a 2-stage arthroplasty is successful in 85% of cases. In our study, 88% of patients demonstrated no clinical evidence of infection for at least 1 year after their revision arthroplasty. This was achieved by eliminating the typical IV drug holiday, reaspiration, and waiting for results. This accelerated the patients return to function and their lives at least 6 weeks sooner. This is particularly noteworthy since this patient population was high risk, and a large percentage had documented chronic osteomyelitis. This necessitated the use of more aggressive surgery such as wide resections and reconstructing with a mega-prosthesis.

The infecting organisms were consistent with the current literature showing that *S aureus* and coagulase-negative *Staphylococcus* were the most common. The culture positivity (69%) was lower than most prior studies (average 8-20% negative). We feel that this may be related to many of the patients being treated with antibiotics prior to our involvement. In the study by Bejon et al., [23] culture-negative infection was surprisingly frequent (41%), despite 93% of cases having at least a 2-week antibiotic-free gap before primary sampling. It may be that the chronic infections referred for 2-stage revisions were more likely to be culture negative. There may also be a failure of organisms to grow on routine culture media because bacteria growing in biofilms simply do not produce colonies when they are transferred to the surfaces of agar plates [24,25]. It is important to note that culture-negative PJIs are especially difficult to diagnose and treat with some being due to *Mycobacteria* and fungi [24,26]. The microbiologic results obtained at reimplantation did not match with those retrieved during the first-stage surgery. Ascione et al. [27] reported positive microbiologic findings during reimplantation 7% (8/114) in patients without continuous antibiotic treatment and 11% (7/82) in patients with antibiotic-free period. There was no microbiologic concordance of bacteria isolated at explant and reimplantation. Bejon et al. [23] and Puhto et al. [28] who employed antibiotic holiday periods had positive cultures on reimplantation at 14% and 5%, respectively. Their microbiologic results were only concordant with only a few patients and were not predictive of an unsuccessful procedure. This is congruent with our findings of 9% positive cultures during reimplantation. However, only 1 patient underwent another surgery within a year. He had a mega-prosthesis and was on SAT. Our return to the operating room in 1 year was 12% which is consistent with previous studies. Of those who required additional surgery, nearly all cases were for en bloc resections who presented with postoperative hematoma/seroma.

Our treatment strategy for antibiotics remained consistent and controlled by a dedicated musculoskeletal infectious disease physician. Following the second-stage reconstruction, we followed the cultures for 2 weeks. If they remained negative, the patient was switched from IV to oral antibiotics and this was continued for 4 weeks. If 2 or more cultures were positive, then the IV antibiotics were continued for 2 weeks postoperatively and were switched to oral antibiotics for another 10 weeks with the option to offer SAT indefinitely. In our study, 40% of the patients were kept on SAT but this population had a large percentage of documented chronic osteomyelitis (greater than or equal to 42%). All patients who were on suppressive antibiotic therapy remained compliant after 1-year follow-up with no incidence of *Clostridium difficile* infection. Eighty-one percent (34/42) of patients who did not undergo surgery within a year had mega-prosthetic reconstructions. SAT may play a role in these high-risk patients, but further studies would need to clarify this.

In standard revisions excluding a mega-prosthesis, we had a 97% (32/33) success rate with only 1 failure who required another surgery. This was consistent with a previous study by Winkler et al. [14] comparing short (<4 weeks) and long interval (≥4 weeks) without antibiotic holiday having similar outcome with 97.4 % infection-free when highly active antibiotic therapy is used. Similarly, among 196 patients, Ascione et al. [27] concluded a favorable outcome rate of 91% in patients who had continuous antibiotic therapy vs 79% in patients who had antibiotic holiday. The authors concluded that continuous antibiotic therapy had a more beneficial outcome among immunocompromised patients because persistent suppression of bacterial growth due to continuous antibiotic therapy reduced low-grade bacterial replication in sanctuaries such as bone sequestration or on the spacer surface. A systematic review by Sabater-Martos et al. [29] including 2387 patients in 24 studies with a global failure rate of 18% found that there is a similar failure rate when reimplantation cultures are negative with or without antibiotic holidays. However, there is a higher risk of failure in those culture positive patients in the holiday group (odds ratio [OR]: 4.798) than in the nonholiday group (OR: 2.225). Reimplantation can be considered without an antibiotic-free period, with additional antibiotics before and after reimplantation even in high-risk patients with standard revisions or those who require endoprosthesis reconstructions. We did not rely on ESR and CRP for timing of reimplantation. It has been recommended by the International Consensus Meeting that surgeons should not wait for complete normalization of the inflammatory markers as this may not occur in some patients and/or take a long period of time. Serum inflammatory markers (ESR and CRP) are not believed to be reliable on their own in determining the presence of infection [30].

Our protocol is consistent with the Infectious Disease Society of America recommendation to give IV antibiotics for 6 weeks in between stages [31]. Longer intervals (>8 weeks) in patients with spacers should be avoided because the antibiotic concentration in bone cement decreases and falls below the minimal inhibitory concentrations [12]. The duration of spacer implantation (>100 days) was significantly associated with treatment [32]. In 2-stage exchange arthroplasty, the goal during the prosthesis-free interval is optimal reduction of the pathogen, as well as treatment of soft tissue infection and osteomyelitis. Antimicrobial treatment is not interrupted until the reimplantation. According to Izakovicova et al., “Drug holiday” prior to reimplantation is no longer recommended [12]. During this antibiotic holiday, bacteria may replicate and cause a relapse after reimplantation. A recent meta- analysis by Fraval et al. concluded that there is no proven superiority of an antibiotic holiday during a 2-stage exchange to treat chronic PJI [33]. This practice may subject patients to unnecessary prolongation of their treatment duration without any improvement in outcome. Since there is still a possibility that the new prosthesis is being implanted into a potentially infected area, the antibiotic treatment is continued after reimplantation for additional 6 weeks despite negative intraoperative cultures [12]. We give prolonged IV antibiotics for 2 weeks after reimplantation until tissue cultures which are held for 2 weeks are finalized as negative. After that, we deemed it safe to transition to oral antibiotics for another 4 weeks. In institutions where intraoperative tissue cultures are held only for 7 days, patients can be transitioned to oral antibiotics to complete 6 weeks of antibiotics after reimplantation.

As in all studies there are pros and cons. Our patient population may not be typical for the average joint replacement surgeon. Many of our patients were referred by other arthroplasty surgeon’s secondary to the complexity of the patient and/or the surgery that was required. More than 50% of these patients had previously failed other surgical intervention. Therefore, it may be difficult to extrapolate results in a biased population. However, it can certainly be argued that this population may be more difficult to treat and yet our results are equivalent while also having a shortened time interval. We treated all these patients in a multidisciplinary model with a dedicated musculoskeletal infectious disease physician. These patients were seen simultaneously in the office. This can certainly improve outcomes but may not be available to all surgeons. This may make our protocol less reproducible in general practice. A great majority of the surgeries were done by 2 surgeons that could limit its generalizability but arguably made the procedures very consistent by our protocol. The last limitation of our study is the 1-year follow-up. We continue to follow these patients and will hopefully have a better understanding of the success and possible failures.

We were happy to see that our protocol did result in good outcomes in most cases despite prior studies suggesting the contrary. In fact, chronic osteomyelitis and failed prior surgeries have often been associated with less-than-optimal results. Although we did not obtain patient-reported outcomes (perhaps a future investigation), our patients often provided positive feedback and were satisfied with the results. We did spend significant time preoperatively counseling patients. This focused on the treatment being an involved process and preparing them with realistic expectation. Our results also confirmed the distinct possibility that a significant percentage of patients with chronic periprosthetic infection may have chronic osteomyelitis. This can certainly account for the difficulty in eradicating infection in this cohort and a more aggressive surgical approach may help improve outcomes. Of course, that is not without increased perioperative complications and long-term future options.

## Conclusions

Our study suggests that equivalent results and favorable outcomes can be obtained for 2-stage revision arthroplasty with a 6-week protocol vs the more common practice of an extended interval between stage 1 and stage 2 with an antibiotic holiday. We have demonstrated in a high-risk population that infection can be eliminated or controlled for at least 1 year and, in most cases, well beyond that. We treated chronic osteomyelitis like a malignancy and performed wide surgical excision to help reduce recurrence and poor outcomes. By creating a shorter interval for 2-stage revision, system-wide improvements for providers and patients are obtainable. It leads to decreased resource utilization, lower costs, and hopefully happier patients.

Surgeons can follow a reliable practice pattern and have more predictable outcomes, while patients may experience reduced pain and less psychosocial burden associated with undergoing a revision. Anecdotally, we have seen that the interval period is a time of increased pain and stress for patients. It is during this time that their limited mobility, difficulties performing activities of daily living and time out-of-work make them frustrated and often accentuates their pain. Therefore, the goal of creating a shorter interval is to get patients back to their preinfection baseline as quickly as possible without compromising efficacy of the procedure.

## CRediT authorship contribution statement

**Alexander Mass:** Writing – review & editing, Writing – original draft. **Myrla Sajo:** Writing – review & editing, Writing – original draft. **Eric Silverstein:** Writing – review & editing, Writing – original draft, Supervision, Formal analysis, Conceptualization.

## Conflicts of interest

E. Silverstein is a paid consultant for Urgo Medical and is an MSIS oncology associate editor for Techniques in Orthpaedics; all other authors declare no potential conflicts of interest.

For full disclosure statements refer to https://doi.org/10.1016/j.artd.2026.101987.

## Appendix A Supplementary Data

Conflict of Interest Statement for Mass

Conflict of Interest Statement for Sajo

Conflict of Interest Statement for Silverstein

## References

[1] Projected volume of primary total joint arthroplasty in the U.S., 2014 to 2030. https://oce-ovid-com.libraryproxy.quinnipiac.edu/article/00004623-201809050-00003/HTML.

[2] Schwartz A.M., Farley K.X., Guild G.N., Bradbury T.L. (2020). Projections and epidemiology of revision hip and knee arthroplasty in the United States to 2030. J Arthroplasty.

[3] Premkumar A., Kolin D.A., Farley K.X., Wilson J.M., McLawhorn A.S., Cross M.B. (2021). Projected economic burden of periprosthetic joint infection of the hip and knee in the United States. J Arthroplasty.

[4] Charette R.S., Melnic C.M. (2018). Two-stage revision arthroplasty for the treatment of prosthetic joint infection. Curr Rev Musculoskelet Med.

[5] Lazic I., Scheele C., Pohlig F., von Eisenhart-Rothe R., Suren C. (2021). Treatment options in PJI – is two-stage still gold standard?. J Orthop.

[6] Lentino J.R. (2003). Prosthetic joint infections: bane of orthopedists, challenge for infectious disease specialists. Clin Infect Dis.

[7] Leone S., Borrè S., Monforte A.A., Mordente G., Petrosillo N., Signore A. (2010). Consensus document on controversial issues in the diagnosis and treatment of prosthetic joint infections. Int J Infect Dis.

[8] Kuzyk P.R.T., Dhotar H.S., Sternheim A., Gross A.E., Safir O., Backstein D. (2014). Two-stage revision arthroplasty for management of chronic periprosthetic hip and knee infection: techniques, controversies, and outcomes. J Am Acad Orthop Surg.

[9] Franceschini M., Pedretti L., Cerbone V., Sandiford N.A. (2022). Two stage revision: indications, techniques and results. Ann Joint.

[10] Kurapatti M., Oakley C., Singh V., Aggarwal V.K. (2022). Antibiotic therapy in 2-Stage revision for periprosthetic joint infection: a systematic review. JBJS Rev.

[11] Aalirezaie A., Abolghasemian M., Busato T., Dennis D., Ghazavi M., Holst D.C. (2019). Hip and knee section, treatment, two-stage exchange: proceedings of international consensus on orthopedic infections. J Arthroplasty.

[12] Izakovicova P., Borens O., Trampuz A. (2019). Periprosthetic joint infection: current concepts and outlook. EFORT Open Rev.

[13] Zimmerli W., Trampuz A., Ochsner P.E. (2004). Prosthetic-joint infections. New Engl J Med.

[14] Winkler T., Stuhlert M.G.W., Lieb E., Müller M., von Roth P., Preininger B. (2019). Outcome of short versus long interval in two-stage exchange for periprosthetic joint infection: a prospective cohort study. Arch Orthop Trauma Surg.

[15] Lum Z.C., Holland C.T., Meehan J.P. (2020). Systematic review of single stage revision for prosthetic joint infection. World J Orthop.

[16] Klouche S., Leonard P., Zeller V., Lhotellier L., Graff W., Leclerc P. (2012). Infected total hip arthroplasty revision: One- or two-stage procedure?. Orthop Traumatol Surg Res.

[17] Leonard H.A.C., Liddle A.D., Burke Ó., Murray D.W., Pandit H. (2014). Single- or two-stage revision for infected total hip arthroplasty? A systematic review of the literature. Clin Orthop Relat Res.

[18] Tibrewal S., Malagelada F., Jeyaseelan L., Posch F., Scott G. (2014). Single-stage revision for the infected total knee replacement: results from a single centre. Bone Joint J.

[19] Haddad F.S., Sukeik M., Alazzawi S. (2015). Is single-stage revision according to a strict protocol effective in treatment of chronic knee arthroplasty infections?. Clin Orthop Relat Res.

[20] Srivastava K., Bozic K.J., Silverton C., Nelson A.J., Makhni E.C., Davis J.J. (2019). Reconsidering strategies for managing chronic periprosthetic joint infection in total knee arthroplasty: using decision analytics to find the optimal strategy between one-stage and two-stage total knee revision. J Bone Joint Surg Am.

[21] Parvizi J., Tan T.L., Goswami K., Higuera C., Della Valle C., Chen A.F. (2018). The 2018 definition of periprosthetic hip and knee infection: an evidence-based and validated criteria. J Arthroplasty.

[22] Beam E., Osmon D. (2018). Prosthetic joint infection update. Infect Dis Clin North Am.

[23] Bejon P., Berendt A., Atkins B.L., Green N., Parry H., Masters S. (2010). Two-stage revision for prosthetic joint infection: predictors of outcome and the role of reimplantation microbiology. J Antimicrob Chemother.

[24] Palan J., Nolan C., Sarantos K., Westerman R., King R., Foguet P. (2019). Culture-negative periprosthetic joint infections. EFORT Open Rev.

[25] Ehrlich G.D., DeMeo P., Palmer M., Sauber T.J., Altman D., Altman G. (2012). Culture negative orthopedic biofilm infections [Internet].

[26] Kalbian I., Park J.W., Goswami K., Lee Y.K., Parvizi J., Koo K.H. (2020). Culture-negative periprosthetic joint infection: prevalence, aetiology, evaluation, recommendations, and treatment. Int Orthop (Sicot).

[27] Ascione T., Balato G., Mariconda M., Rotondo R., Baldini A., Pagliano P. (2019). Continuous antibiotic therapy can reduce recurrence of prosthetic joint infection in patients undergoing 2-Stage exchange. J Arthroplasty.

[28] Puhto A.P., Puhto T.M., Niinimäki T.T., Leppilahti J.I., Syrjälä H.P.T. (2014). Two-stage revision for prosthetic joint infection: outcome and role of reimplantation microbiology in 107 cases. J Arthroplasty.

[29] Sabater-Martos M., Boadas L., Trebše R., Grenho A., Sanz-Ruiz P., Marais L.C. (2024). Impact of positive cultures during the second stage of a two-stage exchange: systematic review and meta-analysis. J Arthroplasty.

[30] Parvizi J., Gehrke T. (2018). Proceedings of the second international consensus meeting on musculoskeletal infection. 2018 ICM Proceedings of the Second International Consensus Meeting on Musculoskeletal Infection. https://www.icmortho.org/2018-icm.

[31] Osmon D.R., Berbari E.F., Berendt A.R., Lew D., Zimmerli W., Steckelberg J.M. (2013). Diagnosis and management of prosthetic joint infection: clinical practice guidelines by the infectious diseases Society of America. Clin Infect Dis.

[32] Tan T.L., Kheir M.M., Rondon A.J., Parvizi J., George J., Higuera C.A. (2018). Determining the role and duration of the “Antibiotic Holiday” period in periprosthetic joint infection. J Arthroplasty.

[33] Fraval A., Gould D., Yilmaz M.K., Soriano A., Parvizi J. (2025). Antibiotic holiday in 2-Stage exchange for periprosthetic joint infection: a scoping review. J Bone Joint Surg Am.

